# Risk factors affecting treatment outcomes for pulmonary tuberculosis in Finland 2007–2014: a national cohort study

**DOI:** 10.1186/s12889-020-09360-7

**Published:** 2020-08-17

**Authors:** Virve Korhonen, Outi Lyytikäinen, Jukka Ollgren, Hanna Soini, Tuula Vasankari, Petri Ruutu

**Affiliations:** 1 Department of Health Security, Finnish Institute for Health and Welfare, Helsinki, Finland; 2 Department of Respiratory Medicine, Tampere University Hospital, Tampere, Finland; 3 Faculty of Medicine and Health Technology, Tampere University, Tampere, Finland; 4 Finnish Lung Health Association (Filha), Helsinki, Finland; 5 Faculty of Medicine, University of Turku, Turku, Finland

**Keywords:** Treatment outcome, Treatment, Mortality, Cohort analysis, Surveillance, Tuberculosis

## Abstract

**Background:**

Major transition in tuberculosis (TB) epidemiology is taking place in many European countries including Finland. Monitoring treatment outcome of TB cases is important for identifying gaps in the national TB control program, in order to strengthen the system. The aim of the study was to identify potential risk factors for non-successful TB treatment outcomes, with a particular focus on the impact of comorbidities. We also evaluated the treatment outcome monitoring system.

**Methods:**

All notified microbiologically confirmed pulmonary TB cases in Finland in 2007–2014 were included, except multi-drug resistant (MDR) cases. Nationwide register data were retrieved from: Infectious Diseases Register, Population Register, Cause of Death Register and Hospital Discharge Register. Non-successful outcomes were divided into three groups: death, unsatisfactory outcomes and non-defined outcomes. Logistic regression analyses were used to identify risk factors for non-successful outcomes.

**Results:**

Treatment outcomes were notified for 98.6% of study cases (*n* = 1396/1416). Treatment success rate was 75%. The main reason for non-successful outcome was death (16%), whereas outcomes failed and lost to follow-up were rare (1% together). In a multivariable model, risk factors for death as outcome were increasing age, male gender and Charlson comorbidity index ≥1, for unsatisfactory outcomes non-MDR drug resistance and TB registered in the first study period, and for non-defined outcomes non-MDR drug resistance. Among 50 cases with unsatisfactory outcomes, we observed false outcome allocations in eight (16%), and > 2% of the cases transferred to another country or disappeared before or during treatment.

**Conclusions:**

With a high proportion of older population among tuberculosis cases, death is a common treatment outcome in Finland. Comorbidity is an important factor to be incorporated when interpreting and comparing outcome rates. There was a considerable inconsistency in outcome allocation in the monitoring system, which implies that there is need to review the guidelines and provide further training for outcome assessment.

## Background

Adequate TB treatment is important both for the recovery of the patient and prevention of transmission, together with early diagnoses and appropriate contact investigations. Tuberculosis treatment outcome monitoring is essential for evaluating the function of a national tuberculosis control program. European Centre for Disease Prevention and Control (ECDC) [1] and World Health Organization (WHO) [2] recommend assessing outcomes for all TB cases with different criteria and timing for drug-susceptible and multidrug-resistant (MDR) TB. Recommendations by ECDC for European Union/European Economic Area (EU/EEA) countries are consistent with WHO recommendations, except that the outcomes are assessed at the latest at 12 months after treatment initiation and hence an outcome group of ‘still on treatment’ is included [1].

Treatment success rates in most European countries do not reach the global target of 85% set by WHO, varying widely from 56% in Hungary to 89% in Norway during 2002–2011 [3]. In 2015, WHO has set more ambitious targets for treatment outcome, including success of at least 90% by year 2025, as well as reducing tuberculosis deaths by 75% [4]. In routine outcome monitoring from European region countries for cases registered in 2016, treatment success rate was 75% and proportion of fatal cases was 8% [1].

In Finland, with a current TB incidence rate of 4.1/100000, the incidence among Finnish-born has decreased and immigration from high-TB-incidence countries has increased raising the proportion of TB cases with foreign origins from 4% in 1995–1996 [5] to 40% in 2017 [6]. The majority of TB among the Finnish-born population is in the elderly persons from reactivation of a latent TB infection, reflected by the mean age of 71 years among Finnish-born TB cases in 2017, the highest in the EU/EEA countries [1]. In countries with a high proportion of elderly cases, comorbidities are common, probably contributing to higher mortality observed in TB outcome monitoring [7] and complicating the interpretation of treatment non-success rates between countries.

A previous cohort study of culture-confirmed pulmonary TB cases registered in 1995–1996 in Finland showed that there was a wide variety of TB treatment combinations and durations [5]. Treatment success rate was 65, and 19% of cases died before or during TB treatment [5]. Thereafter, a National TB Control Program has been published, with recommendations for TB treatment and the use of directly observed treatment (DOT) for risk groups in 2006 [8] and for all patients in 2013 [9].

The aim of our study was to identify potential risk factors for non-successful TB treatment outcomes, with a particular focus on the impact of comorbidities. We also evaluated the treatment outcome monitoring system in order to identify ways to improve the treatment success rate, and to strengthen the TB monitoring program in the changing epidemiologic environment.

## Methods

### Data sources

**Fig. 1:**
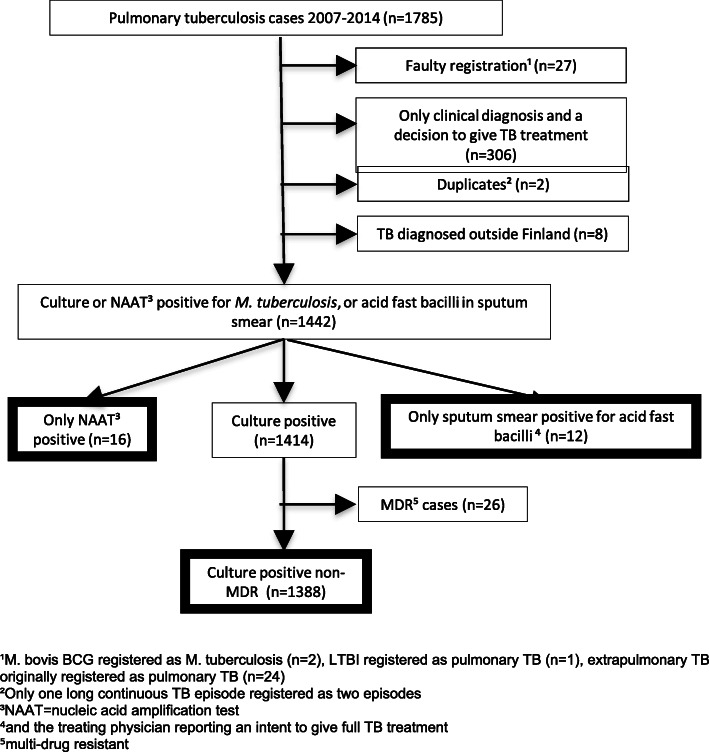
Steps in identifying cases for treatment outcome evaluation, Finland 2007–2014

Our cohort study was based on the data of National Infectious Diseases Register (NIDR), National Population Register, Cause of Death Register and Finnish Hospital Discharge Register. This study included all microbiologically confirmed pulmonary TB cases, except MDR-TB cases, notified from January 1, 2007, to December 31, 2014, to the National Infectious Disease Register (NIDR) (Fig. 1).

Reporting of TB cases to NIDR is mandatory to all physicians and laboratories, and laboratories send an automated reminder to the treating physician of the obligation to notify, when TB is microbiologically confirmed. The date of death is retrieved from the national population registry and HIV cases are linked to TB cases in NIDR. Data from the different sources are automatically linked as a case by the unique national identifier. Following data was collected from NIDR: patient identification (name and unique national identifier), age, gender, origin (country of birth or if unknown, nationality), place of residence, a history of earlier TB diagnosis (since 1950) and its treatment (year, did the patient receive (full) treatment), ICD10 codes (International Classification of Diseases, 10th Revision), microbiological test results including the results of drug susceptibility testing in the national reference laboratory, date of registration in NIDR, and the intent to give full TB treatment.

**Fig. 2:**
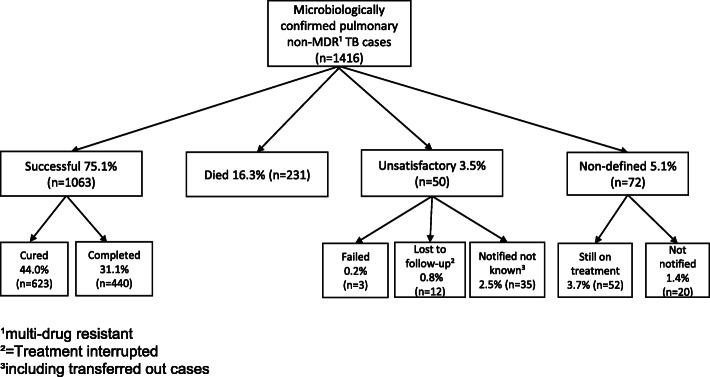
Treatment outcomes in 1416 microbiologically confirmed pulmonary non-MDR TB cases, 2007–2014

Since 2007, treatment outcomes for all microbiologically confirmed pulmonary TB cases in Finland are notified to NIDR according to the WHO/IUATLD recommendation for EU/EEA countries [10] with two additional categories ‘Still on treatment’ [1] and ‘Notified not known’ (Fig. 2). At 12 months from the date of registration, NIDR sends a request for outcome notification to the hospital district (*n* = 20), from which the TB case was originally notified. Outcome notification includes data on treatment of the current TB episode (dates on treatment commencement and cessation, total duration of treatment interruptions, duration of rifampicin plus isoniazid given concomitantly), the patient moving to another hospital district during treatment and the district to which the case moves. One trained physician notifies all outcomes in each hospital district. For cases with outcomes failed, lost to follow-up and notified as not known, we acquired additional information from the notifying health care facility to assess whether the outcomes had been notified correctly.

For cases with death as outcome, we obtained the structured data on the causes of death from the Cause of Death Register at Statistics Finland, in order to determine whether death was TB-related. A death was classified as TB-associated, if TB was the immediate or underlying cause of death or a significant condition contributing to death.

To assess comorbidities, all diagnoses, coded in ICD-10 for study cases, were obtained from National Hospital Discharge Register [11] three years preceding and one year following TB registration date. Comorbidity scores were calculated using the model developed by Charlson et al. [12]. The Charlson-Deyo [13] comorbidity index contains 17 diagnostic groups for chronic diseases with a weighted score that is associated with 1-year mortality, and the model has been used with administrative register data, and with ICD-10 diagnostic codes [14].

### Analysis and statistics

According to the WHO criteria [2], successful treatment outcome includes cases with completed treatment, with or without microbiological evidence of cure. For statistical analysis, we combined failed cases, with a positive culture or sputum smear at 5 months or later, cases with outcomes lost to follow-up (treatment interrupted) and notified not known, including transferred out cases, as a group of unsatisfactory outcome (Fig. 2). Cases with outcomes still on treatment and not notified were combined as a group of non-defined outcome. Death as outcome includes also cases diagnosed post mortem (e.g. at autopsy).

The allocation of each case in outcome grouping in Fig. 2 is based on the original routine outcome notification. For the study, patient chart review was performed for cases with outcomes failed, lost to follow-up and notified not known. We used 5.5 months as the minimum duration for full treatment, as described in our previous study [5]. We used logistic regression analyses for modelling the outcome of tuberculosis treatment. Apart from well-known risk factors, we incorporated in the multivariable analysis the variables with a 20% likelihood in the univariate analysis, as well as the Charlson comorbidity score. We used multi-imputation of the missing data of some variables with the MICE algorithm [15] assuming that the missing values were randomly distributed (MAR). In the model, the shape of the variable age was selected using Akaike information criteria (AIC) [16] because of the high risk of death among the very young children (J-shaped curve). We found that the inverse of age (1/age) was a parsimonious way of modelling the young age effect on the mortality. Furthermore, we incorporated into the model the variables with interactions with age, for which statistical evidence was seen with e.g. AIC. Due to possible clustering in hospital district level, robust standard error estimators were used in the model.

## Results

Of the 1785 pulmonary TB cases notified during 2007–2014, we identified 1416 bacteriologically confirmed non-MDR cases (Fig. 1). The proportion of males was 64.6% and those of foreign origin 27.3%. Three most frequent countries of birth among foreign-born cases were Somalia, Vietnam and countries of the former Soviet Union. The median age of cases was 60 years (range, 0–98 years). Finnish-born cases were significantly older (median age 70 vs. 28 years, *p* < 0.001) and had more comorbidities (Charlson comorbidity index> 0 in 61.9% vs. 22.1%, p < 0.001) than cases with foreign origins. Of the *M. tuberculosis* isolates, 89.1% were fully susceptible. The proportions of mono-resistant and non-MDR poly-resistant isolates were 6.5 and 1.8%, respectively. Twenty-five cases (1.8%) were HIV positive.

### Treatment outcomes and treatment duration

Treatment outcome was notified for 1396 cases (98.6%) (Fig. 2). Successful treatment outcome was achieved in 1063 cases (75.1%). When calculated for only cases with a notified outcome, treatment success rate was 76.1%. The main reason for non-successful outcome was death in 231 cases (16.3%). Outcomes ‘failed’ and ‘lost to follow-up’ together accounted for 15 cases (1.0%).

Among the 1204 cases with data on treatment duration, median duration was 204 days (interquartile range (IQR), 183–278 days). Among cases notified as successfully treated and with data on treatment duration (*n* = 1010/1063), 6 cases (0.6%) received treatment for less than 5.5 months, 913 cases (90.4%) for 5.5 months to one year, and 91 cases (9.0%) for more than a year. Among cases with outcome lost to follow-up (treatment interrupted) and with data on treatment duration (n = 10/12), one (10%) did not receive any medication, five (50%) received treatment for less than 5.5 months, three (30.0%) for 5.5 months to one year and one (10%) for more than one year. Data on the duration of pauses in treatment was available for 773 (64.2%) of the 1204 cases. Among these, TB treatment was discontinued for at least one day for 242 cases (31.3%) and the mean duration of the sum of pauses was 6.7 days per patient.

### Fatal cases

The proportions of death as outcome for Finnish-born and foreign-born cases were 21.8 and 1.6%, respectively. The median time from the date of registration to death was 44 days (IQR, 12–121 days). Among the 150 cases who received TB medication, with data on treatment duration, 81 cases (54.0%) received medication for less than 2 months, 56 (37.3%) for two to less than 5.5 months, 12 (8.0%) for 5.5 months to a year and one (0.7%) for more than a year. Fifty-eight (25.1%) fatal cases did not receive any TB medication.

According to death certificates, death was classified as TB-associated in 167 fatal cases (72.3%); in 101 (43.7%) TB was as the immediate or underlying cause of death, and in 64 (28.6%) as a significant condition contributing to death. Charlson comorbidity scores for cases with TB-associated death did not differ from scores for non-TB-associated deaths (*p* = 0.13).

In univariate analysis, risk factors for death were increasing age, male gender, Finnish origin and Charlson comorbidity index ≥1 (Table 1). In multivariate logistic regression model, independent association for death as outcome was observed with increasing age, male gender and Charlson comorbidity index (Table 2). Interaction was observed between age and gender. The association of higher Charlson index with increased risk for death was seen in all age groups (data not shown).

**Table 1:** Univariate analysis for risk factors for non-successful outcomes in 1416 pulmonary non-MDR TB cases

**Variable**	**Category**	**Successful** **(*n*** **= 1063)**	**Died** **(*n*** **= 231)**	**Univariate RRR**^**a**^ **for death**^**b**^ **(95%CI)** **p**	**Unsatisfactory**^**c**^ **(*n*** **= 50)**	**Univariate RRR**^**a**^ **for unsatisfactory**^**b**^ **(95%CI)** **p**	**Non-defined** **(*n*** **= 72)**	**Univariate RRR**^**a**^ **for** **non-defined**^**b**^ **(95%CI)** **p**
**Age**	Age/10 years	Median 57 years	Median 79 years	1.77 (1.53–2.06) **< 0.001**	Median 27.5 years	0.65 (0.59–0.72) **< 0.001**	Median 51.5 years	0.96 (0.87–1.05) 0.341
**Gender**	Female (*n* = 501)	*n* = 387 (77.2%)	*n* = 68 (13.6%)	1	*n* = 17 (3.4%)	1	*n* = 29 (5.8%)	1
	Male (*n* = 915)	*n* = 676 (73.9%)	*n* = 163 (17.8%)	1.37 (1.06–1.77) **0.015**	*n* = 33 (3.6%)	1.11 (0.68–1.82) 0.677	*n* = 43 (4.7%)	0.85 (0.49–1.46) 0.552
**Origin**	Finnish (*n* = 1030)	*n* = 744 (72.2%)	*n* = 225 (21.8%)	1	n = 13 (1.3%)	1	*n* = 48 (4.7%)	1
	Foreign (*n* = 386)	*n* = 319 (82.6%)	n = 6 (1.6%)	0.06 (0.04–0.11) **< 0.001**	*n* = 37 (9.6%)	6.64 (3.68–11.98) **< 0.001**	*n* = 24 (6.2%)	1.17 (0.70–1.93) 0.551
**Drug resistance non-MDR**^d^	No (*n* = 1262)	*n* = 966 (76.5%)	*n* = 207 (16.4%)	1	n = 38 (3.0%)	1	*n* = 51 (4.0%)	1
	Yes (*n* = 118)	n = 74 (62.7%)	n = 12 (10.2%)	0.76 (0.33–1.74) 0.511	n = 12 (10.2%)	4.12 (2.02–8.41) **< 0.001**	n = 20 (16.9%)	5.12 (3.23–8.11) **< 0.001**
**Sputum smear**^e^	Negative (*n* = 629)	*n* = 481 (76.5%)	n = 96 (15.3%)	1	n = 23 (3.7%)	1	n = 29 (4.6%)	1
	Positive (*n* = 694)	*n* = 527 (75.9%)	*n* = 105 (15.1%)	0.998 (0.82–1.21) 0.986	n = 24 (3.5%)	0.95 (0.48–1.87) 0.888	n = 38 (5.5%)	1.20 (0.80–1.79) 0.387
**Study period**	2007–2010 (*n* = 760)	*n* = 570 (75.0%)	n = 118 (15.5%)	1	n = 31 (4.1%)	1	*n* = 41 (5.4%)	1
	2011–2014 (*n* = 656)	*n* = 493 (75.2%)	*n* = 113 (17.2%)	1.11 (0.88–1.39) 0.383	*n* = 19 (2.9%)	0.71 (0.51–0.99) **0.045**	n = 31 (4.7%)	0.87 (0.43–1.77) 0.709
**Charlson**^f^	0 (*n* = 631)	*n* = 550 (87.2%)	*n* = 30 (4.8%)	1	n = 17 (2.7%)	1	*n* = 34 (5.4%)	1
	1–2 (*n* = 462)	*n* = 322 (69.7%)	*n* = 111 (24.0%)	6.32 (4.57–8.74) **< 0.001**	n = 9 (2.0%)	0.90 (0.30–3.73) 0.859	n = 20 (4.3%)	1.00 (0.60–1.67) 0.985
	3–4 (*n* = 172)	*n* = 109 (63.4%)	*n* = 54 (31.4%)	9.08 (5.63–14.66) **< 0.001**	n = 1 (0.6%)	0.30 (0.04–2.36) 0.251	*n* = 8 (4.7%)	1.19 (0.50–2.82) 0.697
	≥5 (*n* = 85)	n = 52 (61.2%)	*n* = 28 (32.9%)	9.87 (4.63–21.06) **< 0.001**	n = 1 (1.2%)	0.62 (0.11–3.67) 0.600	n = 4 (4.7%)	1.24 (0.55–2.79) 0.596

^a^ratio of relative risks

^b^compared to successfully treated cases

^c^ including cases transferred out

^d^Information missing for 36 cases (23 successfully treated cases, 12 fatal cases, 1 non-defined case)

^e^Information missing for 93 cases (55 successfully treated cases, 30 fatal cases, 3 other defined unsuccessfully treated cases, 5 non-defined outcome)

^f^Information missing for 66 cases (30 successfully treated cases, 8 fatal cases, 22 other defined unsuccessfully treated cases, 6 non-defined outcome)

**Table 2:** Multivariable analysis for risk factors for death in 1416 pulmonary non-MDR TB cases

**Variable**	**Multivariable RRR**^**a**^ **for death (95% CI)**	**p**
**Age/10 years**^b^		
**Finnish male**	1.50 (1.25–1.80)	**< 0.001**
**Finnish female**	1.94 (1.42–2.65)	**< 0.001**
**Foreign origin male**	1.20 (0.92–1.57)	0.183
**Foreign origin female**	1.55 (1.04–2.30)	**0.030**
**Male**	9.54 (1.36–66.70)	**0.023**
**Foreign origin**	1.01 (0.20–5.21)	0.991
**Drug resistance non-MDR**^c^	0.84 (0.33–2.11)	0.705
**Study period 2011–2014**	1.06 (0.80–1.40)	0.695
**Charlson 1–2**^d^	3.03 (2.11–4.35)	**< 0.001**
**Charlson 3–4**^d^	3.55 (2.20–5.75)	**< 0.001**
**Charlson ≥ 5**^d^	5.94 (2.65–13.33)	**< 0.001**

^a^ratio of relative risks

^b^1/age was also included in the model to explain the increase in the

risk of death among very young children (curve J-shaped), *p* < 0.001.

^c^Information imputed for 23 successfully treated cases, 12 fatal cases

^d^Information imputed for 30 successfully treated cases, 8 fatal cases

### Unsatisfactory outcomes

Among the 12 cases with outcome lost to follow-up, premature treatment cessation was due to an adverse reaction in three cases, non-compliance because of substance abuse in four, and in one, a positive TB culture had been missed. Among the 35 cases notified as outcome not known, 33 cases were of foreign origin. Out of the 35, 25 transferred to another country before or during TB treatment, three transferred within Finland and four cases disappeared. Among the 50 cases with unsatisfactory outcomes, we observed false outcome allocations in eight cases (16%).

In univariate analysis, risk factors for unsatisfactory outcome (failed, lost to follow-up and notified not known) were younger age, foreign origin, non-MDR drug resistance and TB registered in the first study period (years 2007–2010) (Table 1). In multivariable logistic regression model, an independent association was observed with non-MDR drug resistance (ratio of relative risks (RRR), 2.6; 95% confidence interval (CI), 1.2–5.8) and TB registered during the first study period (RRR, 1.5; 95%CI, 1.1–2.1). Interaction was observed between age and origin. When we restricted analysis to only outcomes ‘failed’ and ‘lost to follow-up’ combined, we found that non-MDR drug resistance was a clear predictor for these outcomes (univariate RRR, 4.75; 95%CI, 2.05–11.02, multivariate RRR, 4.93; 95%CI, 2.26–10.74).

### Non-defined outcomes

Among the 52 cases with outcome still on treatment at 12 months, the reasons for extension of treatment was reported for 36 cases (69%) and were following: having non-MDR drug resistant isolate (*n* = 19), miliary/disseminated disease (*n* = 5), advanced cavitary disease (*n* = 4), pauses on treatment because of adverse drug reactions (*n* = 4), bone and joint tuberculosis (*n* = 2), recurrent TB (*n* = 1) and prolonged sputum culture positivity (*n* = 1). Only non-MDR drug resistance was associated with non-defined outcome in univariate (Table 1) and multivariable regression analysis (RRR, 5.6; 95%CI, 3.8–8.1).

### Quality of treatment outcome allocations and treatment follow-up

Based on the patient chart review and additional information in the notifications of the 50 cases with an unsatisfactory outcome, we observed false outcome allocations in eight cases (16%): four among cases with outcome lost to follow-up, three among cases with outcome not known and one among cases with outcome failed. Among these eight, two should have been categorized as successful outcome, two still on treatment, one not known (transferred) and three lost to follow-up, including one refusal. According to the additional data mentioned above, 26 (1.8% of all) cases transferred to another country and seven (0.5%) cases disappeared before or during TB treatment. Among the 31 cases who were originally notified as not known (transferred to another hospital district in Finland during TB treatment), the final treatment outcome was notified for 28 (90.3%) (2 still on treatment, 1 died, 25 successful). Among cases notified as successfully treated and with data on treatment duration (1010/1063, 95%), 91 cases (9.0%) had received treatment for more than a year according to the outcome notification.

## Discussion

Our population-based cohort study on risk factors for non-successful treatment outcomes of 1416 pulmonary TB cases during 2007–2014 in Finland indicates that comorbidities, in addition to age and male gender, have an independent contribution to death as outcome. Death constituted two thirds of the non-successful outcomes and was the main reason for the proportion of successful outcomes remaining far below the 85% success rate objective set by the WHO.

Although there has been a minor decline in death as outcome in pulmonary TB in Finland from 19% in 1995–1996 [5] to current 16%, the proportion of death as outcome is still remarkably higher than the overall 7 % found in the 16 EU/EEA countries in 2002–2011 [3]. However, this is in line with some other European countries, e.g. Czech Republic (18%) and Slovenia (14%), with similar demographics among TB patients [17, 18]. Earlier studies have shown several non-infective comorbidities, e.g. diabetes [19, 20], liver [21] and kidney disease [19–21] as well as COPD [19], to be associated with death as outcome, but the combined effect of comorbidities on death with TB has rarely been studied [7]. In a case-control register study from Denmark [7], comorbidities were shown to be associated with death, and mortality among TB cases was significantly higher than among matched controls for all age groups above twenty years. With a median age as high as 70 years among Finnish-born patients, comorbidities are common. Using the Charlson comorbidity index [12, 14] we found an association between comorbidity and the risk of death; this was seen in all age groups. Beside the fact that comorbidities may directly cause death in a patient with TB, they also may cause delays in TB diagnostics by offering alternative explanations for patients’ symptoms. In a study from the US, tuberculosis-related deaths were associated with patients having an alternative diagnosis, e.g. pneumonia, before TB diagnosis [22]. In our study, two thirds of fatal cases with data on TB treatment duration died before treatment was started or received treatment for less than two months (early deaths with TB). This is in line with previous studies from California [23] and North Carolina [24], emphasizing that TB diagnosis is often missed or delayed with current diagnostic tools. We did not find a clear difference in Charlson comorbidity scores between cases with TB-associated and non-TB-associated death according to data on death certificates. It has been stated that the diagnostic accuracy of death certificate data is often poor [25, 26], especially when death takes place outside hospital [27].

We found a striking difference between Finnish-born cases and those with foreign origins: over one-fifth of Finnish-born cases versus less than 2 % of those with foreign origins had death as outcome, but origin was not independently associated with death in multivariable analysis. Male gender was a risk factor for death as outcome, consistent with our earlier study in Finland [28] as well as previous reports from other low-incidence countries [3, 7, 29]. We were not able to evaluate reasons for the large risk difference between genders particularly at young age groups, because we did not have data on several risk factors, such as alcohol and substance abuse, the use of immunosuppressive medications and the implementation of directly observed therapy (DOT). Alcohol abuse has been associated with death among TB cases in several studies [23, 24, 30] and especially among patients younger than 65 years [31].

Outcomes ‘failed’ and ‘lost to follow-up’ combined accounted for only 1% of outcomes in our study, which is far less than the 8% found in the 16 EU/EEA countries in 2002–2011 [3]. We observed that non-MDR drug resistance was a risk factor for outcomes ‘failed’ and ‘lost to follow-up’ combined, as well as for the group of unsatisfactory outcomes, which includes also cases with outcome notified as not known. Isoniazid resistance has been described as a risk factor for non-successful treatment, including also fatal cases, in earlier studies in low-incidence countries [32, 33], but this finding has been inconsistent [34, 35]. Our finding underscores the need for training physicians and having a special focus in the national guidelines on appropriate treatment of drug resistant cases and the importance of routine pheno- and genotypic drug-susceptibility testing. Even though our study period did not include the year 2015, when large numbers of asylum seekers arrived Finland, more than 2% of cases transferred to another country or disappeared before or during TB treatment. This results at a high risk of treatment interruption, and the potential for continued transmission, and stresses the need to develop a system to inform the known or probable receiving country when a TB patient emigrates or disappears.

When evaluating the quality of treatment outcome notifications, we observed that 16% of cases originally notified with an unsatisfactory outcome had been misclassified. Furthermore, almost 10% of cases notified as successfully treated, whose treatment duration was reported in the outcome notification, should have been categorized as still on treatment according to the national guidelines, which sets the evaluation at 12 months from case registration. This suggests that outcome assessment has a considerable rate of deviations from guidelines, even when it is performed by one trained specialist in each hospital district. This, together with our observations on the effect of comorbidity, should be taken into consideration when comparing countries and developing European statistics on TB.

The strengths of this nationally comprehensive cohort study include the observed high reporting rate at almost 99% for pulmonary tuberculosis in the mandatory TB outcome reporting. NIDR sends a request for outcome notification to the hospital district at 12 months from the date of registration, and actively follows up for missing notifications. In addition, the outcome surveillance system was efficient in monitoring transfer of cases within Finland during treatment. We have previously shown a high coverage for the TB surveillance system [36], after which integration of automated mechanisms for laboratory notifications and automated laboratory reminder of obligation to notify TB to the treating physician have been added, ensuring a high coverage and representativeness of the data used. Comorbidities were retrieved from National Hospital Discharge Register, which has a high sensitivity and specificity for the common disease groups [11] constituting a major part of the Charlson comorbidity index.

Limitations of our study include the fact that we did not have data on some known risk factors, particularly substance abuse. Furthermore, we did not have data on concomitant severe extrapulmonary TB manifestations, such as meningeal TB. Until 2013, DOT was recommended in national guidelines [8] for only certain risk groups, e.g. alcohol or substance abuse, elderly patients and patients with many comorbidities, but the absence of data on DOT use is unlikely to introduce a major bias on our analysis. Additionally, Charlson comorbidity index does not contain all disease groups, which may influence TB treatment outcome, such as inflammatory bowel disease and vasculitis, in which immunosuppressive medication is commonly used. Less than 2% of TB cases were HIV positive, and as a country with a very low incidence for HIV [6], the absence of HIV test results is unlikely to introduce a bias. Treatment outcomes for solely clinically diagnosed TB cases have been notified only since year 2015 in Finland.

## Conclusions

With a high proportion of older population among TB cases, death is a common treatment outcome in Finland. Comorbidities contribute to death with TB in all age groups, and two thirds of deaths occur within two months after TB registration. Increased awareness of TB as well as a low threshold for TB suspicion and diagnostics is needed. In addition, early empiric TB treatment when the suspicion of TB is strong and sufficient material for TB diagnostics have been collected, as well as adequate treatment of comorbidities during TB treatment should be emphasized. Moreover, a special focus should be given in the national guidelines on the treatment of non-MDR drug resistant cases, as these are at risk for other non-successful outcomes than death. Furthermore, as we identified errors in outcome allocation, there is a need to review guidelines and provide further training in outcome assessment.

## Data Availability

The data sources of this study were National Infectious Diseases Register (https://thl.fi/fi/web/infektiotaudit-ja-rokotukset/seurantajarjestelmat-ja-rekisterit/tartuntatautirekisteri) and Finnish Hospital Discharge Register (https://thl.fi/fi/tilastot-ja-data/ohjeet-tietojen-toimittamiseen/hoitoilmoitusjarjestelma-hilmo), maintained by the Finnish Institute for Health and Welfare (www.thl.fi), and Cause of Death Register (https://www.stat.fi/meta/til/ksyyt.html) maintained by Statistics Finland (www.stat.fi). Permissions required in order to access these register data can be applied from the Finnish Institute for Health and Welfare (www.thl.fi). The datasets generated and/or analyzed during the current study are not publicly available due to possibility of recognition of a patient even though data does not include personal level data.

## Abbreviations

TB: Tuberculosis
ECDC: European Centre for Disease Prevention and Control
WHO: World Health Organization
MDR: Multi drug resistant
EU/EEA: European Union/European Economic Area
NIDR: National infectious disease register
ICD10: International Classification of Diseases, 10th Revision
IUATLD: International Union Against Tuberculosis and Lung Disease
AIC: Akaike information criteria
IQR: Interquartile range
RRR: Ratio of relative risks
CI: Confidence interval
DOT: Directly observed therapy.
